# De Ritis (AST/ALT) ratio as a predictor of early adverse outcomes following transcatheter aortic valve replacement

**DOI:** 10.1371/journal.pone.0354267

**Published:** 2026-07-28

**Authors:** Haitham Abu Khadija, Jebrin Alkrinawi, Ali Abdullah, Omar Ayyad, Ammar Barakat, Salma Najjar, Alexander Kogan, Leonid Sternik, Alaa Shehady, Mohammad Masu’d, Duha Najajra, Mohammad Alnees

**Affiliations:** 1 Heart Center, Kaplan Medical Center, Rehovot, affiliated with the Hebrew University, Jerusalem, Israel; 2 Institute of Gastroenterology and Liver Disease, Kaplan Medical Center, Rehovot, Israel, affiliated with the Hebrew University, Jerusalem, Israel; 3 Department of Internal Medicine B, Kaplan Medical Center and Faculty of Medicine, Hebrew University of Jerusalem, Jerusalem, Israel; 4 Department of Cardiac Surgery, The Leviev Cardiothoracic and Vascular Center, Sheba Medical Center, Tel Hashomer, Israel; 5 Gray Faculty of Medical and Health Sciences, Tel Aviv University, Ramat Aviv, Tel Aviv-Yafo, Israel; 6 Harvard Medical School, Postgraduate Medical Education, Global Clinical Scholar Research Training Program, Boston, Massachusetts, United States of America; Second Xiangya Hospital, CHINA

## Abstract

**Background:**

Simple biochemical markers that capture systemic stress could refine short-term risk stratification after TAVR. We evaluated whether an elevated AST/ALT ratio predicts early adverse outcomes following transfemoral TAVR.

**Methods:**

We retrospectively analyzed 733 consecutive patients undergoing transfemoral TAVR. The exposure was the preprocedural AST/ALT ratio categorized as low (<1.4; n = 478) or high (≥1.4; n = 255). Baseline characteristics were compared using t/Wilcoxon tests and χ²/Fisher’s exact tests. Thirty-day endpoints included all-cause mortality, acute kidney injury (AKI), stroke, and new-onset atrial fibrillation (NOAF). Kaplan–Meier failure curves were compared by the log-rank test. Cox models estimated hazard ratios (HRs) with 95% CIs; candidate covariates were prespecified and further refined with LASSO (10-fold cross-validation). Model assumptions were checked with Schoenfeld residuals.

**Results:**

High AST/ALT was associated with greater 30-day risk of AKI (adjusted HR 3.18, 95% CI 1.80–5.63), stroke (adjusted HR 2.73, 95% CI 1.31–5.69), and NOAF (adjusted HR 2.35, 95% CI 1.57–3.51). For mortality, the crude association was not statistically significant (HR 2.07, 95% CI 0.91–4.68; p = 0.082) and remained non-significant after adjustment (HR 1.74, 95% CI 0.75–4.04; p = 0.197). Additional adjusted associations included higher AKI risk with high-intensity statins (HR 3.51, 95% CI 1.75–7.06) and baseline complete right bundle-branch block (HR 3.77, 95% CI 1.49–9.57).

**Conclusions:**

An elevated preprocedural AST/ALT ratio independently identifies TAVR recipients at increased 30-day risk of AKI, stroke, and NOAF, but not mortality. Incorporating this inexpensive marker into routine assessment may help tailor periprocedural strategies and early surveillance after TAVR.

## Introduction

Transcatheter aortic valve replacement (TAVR) has transformed the management of severe aortic stenosis by providing a less invasive alternative to surgical valve replacement across a progressively broader range of patients. Contemporary guidelines have emphasized Heart Team decision-making, procedural standardization, and the central role of dedicated valve centers, reflecting the maturation of TAVR into routine cardiovascular practice with excellent short-term safety and hemodynamic performance [1]. Large national registries further showed durable early outcomes alongside ongoing refinements in vascular access, device technology, and peri-procedural care.

Despite these advances, early complications after TAVR remain clinically important. Within 30 days, stroke, acute kidney injury (AKI), new-onset atrial fibrillation (NOAF), and mortality continue to contribute to disability, prolonged hospitalization, rehospitalization, and worse short-term prognosis [2–7]. Because these events occur during the early recovery window, improved preprocedural identification of higher-risk patients remains an important goal in contemporary TAVR care.

Previous studies in TAVR populations have investigated a range of biomarkers reflecting different domains of physiologic vulnerability, including inflammatory markers (e.g., C-reactive protein [CRP], neutrophil-to-lymphocyte ratio [NLR]), renal indices, metabolic parameters, and nutritional markers. These biomarkers have been associated with early complications and short-term outcomes after TAVR, highlighting the importance of biological risk profiling beyond traditional clinical risk scores [8–13].

A growing body of literature has explored novel biomarkers for preprocedural risk stratification after TAVR, including inflammatory, metabolic, renal, myocardial, and nutritional indices. Such biomarkers may capture multisystem physiologic vulnerability that is not fully reflected by conventional clinical risk scores alone. Although these markers are not themselves therapeutic, earlier recognition of patients at higher biologic risk may have important clinical implications, including closer peri-procedural monitoring, more individualized risk mitigation strategies, and more structured early follow-up, which may ultimately contribute to improved short-term outcomes after TAVR.

Against this background, the aspartate aminotransferase-to-alanine aminotransferase (AST/ALT) ratio is an attractive candidate biomarker because it is inexpensive, routinely available, and biologically linked to systemic stress and organ vulnerability. Prior cardiovascular literature has shown that elevated AST/ALT ratios are associated with adverse outcomes in diverse populations [14–16], but data specifically evaluating this marker in TAVR cohorts remain limited. Importantly, its role as a preprocedural risk marker across multiple early post-TAVR outcomes has not been systematically examined within a unified analytic framework. Accordingly, this study evaluated the preprocedural AST/ALT ratio as a pragmatic laboratory-based marker of early risk after TAVR. Specifically, we examined its association with multiple clinically relevant 30-day outcomes, including stroke, all-cause mortality, NOAF, and AKI, within a unified analytical framework.

## Materials and methods

### Study design and population

This study is a retrospective, longitudinal cohort study that included consecutive patients who underwent transcatheter aortic valve replacement (TAVR) at the Heart Center, Kaplan Medical Center, Israel, between January 2016 and January 2025. Data were accessed for research purposes on 06/07/2025, after institutional Helsinki Committee approval on 13/06/2025 (Kaplan Medical Center, protocol KMC-0045-25).

### Participants

Eligible participants were adults (aged ≥18 years) with severe symptomatic aortic stenosis who underwent transcatheter aortic valve replacement (TAVR) between January 2016 and January 2025 and had complete baseline laboratory data necessary for calculating the aspartate aminotransferase-to-alanine aminotransferase (AST/ALT) ratio. No minors or prisoners were targeted or enrolled.

Of 945 patients who underwent TAVR at Kaplan Medical Center during the study period, 212 were excluded due to deaths occurring within 24 hours after the procedure (n = 25), active malignancy or infection (n = 26), liver cirrhosis (n = 16), extreme AST/ALT values above the 99th percentile (>3.67) (n = 8), or missing baseline AST/ALT results or required laboratory data (n = 137).

**Fig 1:**
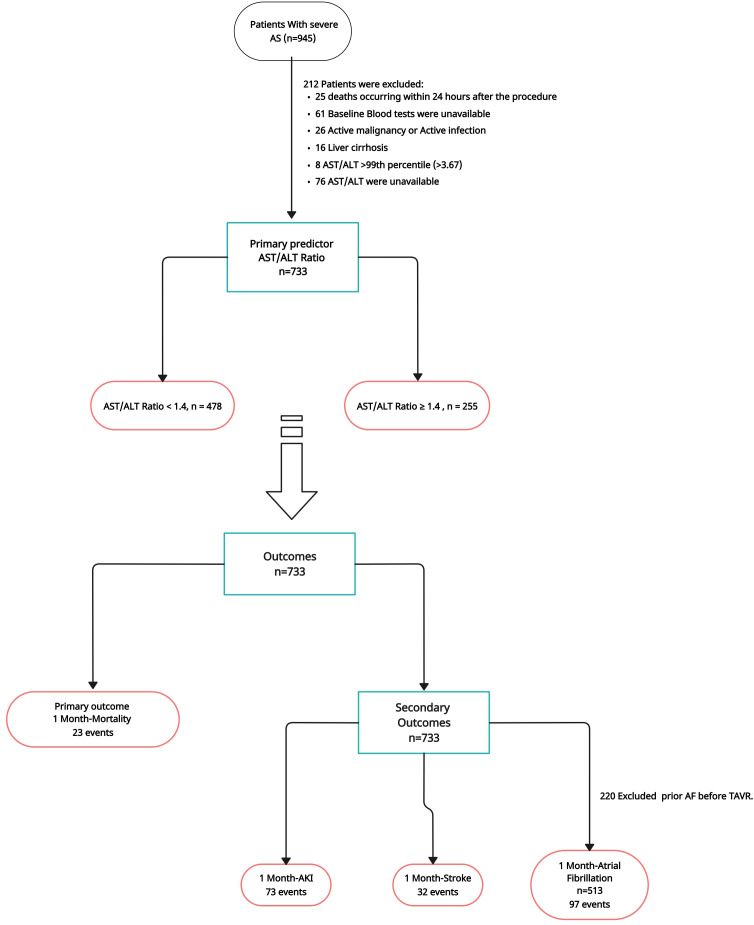
Flowchart of Study. Abbreviations: AS = Aortic Stenosis; AST = Aspartate Aminotransferase; ALT = Alanine Aminotransferase.

The final study cohort included 733 patients, who were categorized according to their baseline AST/ALT ratio into a low-ratio group (<1.4, n = 478) and a high-ratio group (≥1.4, n = 255), as illustrated in Fig 1.

Patients with liver cirrhosis were excluded a priori because advanced chronic liver disease may substantially alter aminotransferase levels independent of cardiovascular risk. In addition, extreme AST/ALT values above the 99th percentile were excluded a priori, prior to outcome analysis, to reduce the influence of atypical or non-cardiac causes of marked enzyme elevation, such as acute hepatic injury or laboratory artifact, and to minimize potential bias related to outlier-driven effects.

Patients who died within 24 hours after the procedure were excluded because these events were predominantly related to acute procedural or mechanical complications (e.g., annular rupture, cardiac tamponade, or major vascular injury) and were considered unlikely to reflect baseline biological vulnerability captured by the AST/ALT ratio.

### Procedural details

All TAVR procedures were performed via the transfemoral route using a dedicated safety-wire technique. Vascular access and closure were achieved with the Prostar XL system (Abbott Vascular, Redwood City, CA, USA). Interventions were conducted in a hybrid operating room under either general anesthesia or, more commonly, local anesthesia with conscious sedation.

Procedural duration was defined as the time from initial femoral arterial puncture (“skin”) to final vascular closure (“skin”). Following vascular access, unfractionated heparin was administered to maintain an activated clotting time (ACT) >250 seconds, and protamine sulfate (up to 50 mg; 1 mg per 100 units of heparin) was administered at the operator’s discretion at the end of the procedure.

In accordance with contemporary ESC recommendations [1], patients received aspirin 100 mg daily or clopidogrel 75 mg daily starting one day before TAVR, unless they were already on chronic antiplatelet or oral anticoagulation therapy, in which case antithrombotic management was individualized. Valve deployment was performed under fluoroscopic guidance, aiming for an implantation depth of 2–3 mm below the annular plane, consistent with current best-practice standards.

### Primary predictor

The primary predictor was the preprocedural AST/ALT ratio. AST and ALT values were obtained from baseline laboratory testing performed before TAVR as part of the routine preprocedural evaluation. The AST/ALT ratio was calculated by dividing the aspartate aminotransferase value by the alanine aminotransferase value. For the primary analysis, patients were categorized into two prespecified groups according to baseline AST/ALT ratio: low (<1.4) and high (≥1.4). The cut-off of 1.4 was selected a priori on the basis of prior cardiovascular literature reporting its prognostic relevance [17].

### Study endpoints

All outcomes were assessed within 30 days after TAVR. The primary endpoint was 30-day all-cause mortality. Secondary endpoints were 30-day AKI, stroke, and NOAF. Outcomes were defined according to VARC-3 criteria and ascertained by review of hospitalization records, laboratory results, procedural reports, discharge summaries, readmission records, and available 30-day follow-up documentation. AKI was classified according to VARC-3 creatinine-based staging criteria using post-procedural serum creatinine measurements obtained during routine clinical care. Stroke events were confirmed from neuroimaging reports and corresponding clinical documentation. NOAF was defined as atrial fibrillation newly documented after TAVR in patients without prior atrial fibrillation and identified from in-hospital rhythm monitoring, electrocardiography, and available post-discharge clinical records.

### Power justification

Post-hoc power calculations were performed using the stpower cox command in Stata. All post-hoc power analyses based on the observed cohort demonstrated excellent statistical strength. Using Cox proportional-hazards modeling with two-sided α = 0.05, the study achieved an estimated power of 1.00 for all evaluated endpoints. Specifically, the analyses showed full power to detect the observed associations for new-onset atrial fibrillation (HR = 2.35), acute kidney injury (HR = 3.18), stroke (HR = 2.73), and all-cause 30-day mortality (HR = 2.07). These findings confirm that the available sample size (N = 733) was sufficient to robustly detect the observed hazard ratios across all primary and secondary outcomes.

### Statistical analysis

All analyses were conducted in Stata/SE 17.0 (StataCorp, College Station, TX). Statistical tests were two-sided with a significance threshold of p < 0.05, and 95% confidence intervals (CIs) are reported throughout. The exposure of interest was the preprocedural AST/ALT ratio, categorized as low (<1.4) versus high (≥1.4).

Baseline characteristics were summarized using means±SD or medians (Q1–Q3) for continuous variables and counts (percentages) for categorical variables, as appropriate. Between-group differences were assessed using t tests or Wilcoxon rank-sum tests for continuous variables (depending on distribution) and χ² or Fisher’s exact tests for categorical variables. These summaries were descriptive and not intended to imply causality.

Time-to-event outcomes within 30 days were analyzed using survival methods. For each endpoint, Kaplan–Meier failure (cumulative incidence) curves were generated by the AST/ALT group with numbers at risk displayed, and groups were compared using the log-rank test to visualize and test for early risk separation.

To estimate adjusted associations, we fitted Cox proportional-hazards models and reported hazard ratios (HRs) with 95% CIs. Candidate covariates were prespecified from prior literature and clinical plausibility [8,18–24]. To reduce overfitting and enhance parsimony given the number of events, we applied the least absolute shrinkage and selection operator (LASSO) with 10-fold cross-validation for each endpoint to identify a predictive subset of variables; multivariable Cox models were then fitted, including the selected predictors (and clinically essential covariates, when applicable).

Continuous analyses were performed by modeling the AST/ALT ratio per 1-standard deviation increase to allow standardized effect estimation across outcomes. This approach was used as a sensitivity analysis to evaluate whether associations were consistent beyond the prespecified categorical threshold. To evaluate the robustness of the primary analysis, a sensitivity analysis was performed including patients with extreme AST/ALT values (>99th percentile), modeling the AST/ALT ratio as a continuous variable per 1-standard deviation increase.

Model assumptions and robustness were rigorously assessed. The proportional-hazards assumption was evaluated using Schoenfeld residuals and visual inspection of log–log survival plots; no meaningful violations were allowed to persist, and time-dependent terms would be considered if needed. Diagnostics included checks for influential observations and collinearity. Individuals with missing event times for a given outcome were excluded from that endpoint’s time-to-event analysis (complete-case approach for the endpoint only). Because AKI ascertainment required post-procedural creatinine measurements, AKI analyses were restricted to patients with the necessary follow-up laboratory data. Reporting followed principles from the TRIPOD guidelines for transparent multivariable modeling [25].

### Ethics statement

This study was approved by the Kaplan Medical Center Institutional Helsinki Committee (KMC-0045-25; committee approval 13/06/2025; director’s authorization 02/07/2025; validity through 02/07/2026). The research used retrospectively collected, de-identified clinical data and posed no more than minimal risk. The requirement for informed consent was waived by the committee because the study could not practically be carried out otherwise. Source data were stored on hospital-owned, access-controlled servers with access restricted to authorized investigators. The study adhered to the Declaration of Helsinki and applicable institutional policies.

## Results

Of the 733 patients included, 255 (34.8%) had a high preprocedural AST/ALT ratio (≥1.4) and 478 (65.2%) a low ratio (<1.4). Baseline demographics and comorbidities were comparable between groups, with a mean age of 81 years, 51% women, and similar rates of diabetes (45%), hypertension (88%), dyslipidemia (80%), and atrial fibrillation (30%) (Table 1). Procedural and echocardiographic parameters were largely balanced, except for a longer median procedural time in the high-ratio group (75 vs 70 minutes, *p* = 0.015) and fewer patients receiving high-intensity statins (40% vs 53%, *p* = 0.006). No significant differences were noted in valve type, annular dimensions, or aortic gradients.

**Table 1:** Baseline and procedural characteristics according to preprocedural AST/ALT ratio.

Variable	AST/ALT < 1.4 (n = 478)	AST/ALT ≥ 1.4 (n = 255)	P value
**Demographics and baseline risk**			
Age, years	80.97 ± 7.14	80.67 ± 7.23	0.600
Female sex, n (%)	253 (52.93%)	129 (50.59%)	0.546
Body mass index, kg/m²	28.17 ± 4.94	28.38 ± 5.27	0.601
Body surface area, m²	1.84 ± 0.21	1.84 ± 0.21	0.976
STS score	6.7 ± 3.5	7.4 ± 3.8	0.417
**Clinical comorbidities**			
Diabetes mellitus, n (%)	217 (45.40%)	116 (45.49%)	0.981
Hypertension, n (%)	426 (89.12%)	224 (87.84%)	0.603
Current or former smoking, n (%)	75 (15.69%)	44 (17.25%)	0.584
Dyslipidemia, n (%)	390 (81.59%)	201 (78.82%)	0.367
Atrial fibrillation, n (%)	145 (30.33%)	75 (29.41%)	0.795
Coronary artery disease, n (%)	216 (45.19%)	100 (39.22%)	0.120
Peripheral vascular disease, n (%)	73 (15.27%)	32 (12.55%)	0.316
**Baseline medications**			
Insulin therapy, n (%)	47 (9.8%)	18 (7.1%)	0.193
Statin use, n (%)	356 (74.5%)	181 (71.0%)	0.338
Oral antidiabetic therapy	147 (63.36%)	85 (36.64%)	0.754
Statin intensity among statin users	(n = 356)	(n = 181)	0.006
Low/moderate intensity, n (%)	168 (47.19%)	108 (59.67%)	
High intensity, n (%)	188 (52.81%)	73 (40.33%)	
**Baseline laboratory variables**			
White blood cell count, K/µL	5.49 ± 3.87	5.37 ± 3.93	0.686
Platelet count, K/µL	194.13 ± 64.39	193.87 ± 77.63	0.962
Total cholesterol, mg/dL	135.51 ± 40.19	137.37 ± 38.73	0.547
Serum creatinine, mg/dL	1.13 ± 0.75	1.17 ± 0.89	0.510
**Baseline echocardiographic variables**			
Septal thickness, mm	12.0 (9.0–13.0)	12.1 (8.0–14.0)	0.187
Left ventricular ejection fraction, %	55.0 (43.9–60.0)	50.0 (43.9–55.0)	0.312
Mean aortic valve gradient, mmHg	54.6 (37–54.6)	54.6 (38–54.6)	0.811
Aortic valve area, cm²	0.71 (0.60–0.81)	0.70 (0.60–0.80)	0.149
**Procedural characteristics**			
Self-expanding valve, n (%)	335 (70.08%)	165 (64.71%)	0.136
Valve size, mm	27.36 ± 2.78	27.39 ± 3.04	0.891
Post-dilatation, n (%)	143 (29.92%)	81 (31.76%)	0.605
Procedural time, min	70 (58–89)	75 (60–95)	0.015
Contrast medium volume, mL	100 (75–125)	100 (70–140)	0.261
**Baseline electrocardiographic and CT variables**			
PR interval, ms	185.76 ± 23.09	187.64 ± 25.64	0.314
QTc interval, ms	441.10 ± 30.52	441.81 ± 25.74	0.751
QRS duration, ms	107.29 ± 27.51	109.57 ± 29.81	0.418
RBBB (complete right bundle branch block)	9 (1.88%)	9 (3.53%)	0.193
Annulus area, mm²	343.30 ± 152.65	345.25 ± 154.49	0.870
Annulus perimeter, mm	59.87 ± 20.39	61.34 ± 18.76	0.341

**Values are presented as mean ± standard deviation (SD), median (Q1–Q3), or n (%) as appropriate. a t test, b Chi-square test (or Fisher’s exact test when appropriate), c Two-sample Wilcoxon rank-sum (Mann–Whitney) test.**

During the 30-day follow-up, 23 deaths (3.1%), 73 AKI events (10.0%), 32 strokes (4.4%), and 97 NOAF cases among patients without prior atrial fibrillation (18.9%; 97/513) were recorded. Among patients who died within 30 days after TAVR, the median time to death was 4 days (IQR 0.1–20 days). A higher AST/ALT ratio was associated with a greater risk of AKI, stroke, and NOAF, but not mortality (Table 2). Additional univariate predictors included older age and longer procedural time for mortality, high-intensity statin therapy for AKI, baseline RBBB for stroke, and oral antidiabetic therapy as a protective factor against stroke.

**Table 2:** Univariate Predictors of 30-Day Mortality, Acute Kidney Injury, Stroke, and NOAF After TAVR.

Variable	Category	TAVR-All cause mortality (n = 733, events = 23)		TAVR-AKI (n = 733, events = 73)		TAVR-Stroke (n = 733, events = 32)		TAVR-NOAF (n = 513, events = 97)	
		**HR (%95 CI)**	**P value**	**HR (%95 CI)**	**P value**	**HR (%95 CI)**	**P value**	**HR (%95 CI)**	**P value**
**Demographic data**									
**Age, y**		**1.100 (1.025–1.180)**	**0.008**	0.994 (0.964–1.025)	0.687	1.00 (0.96–1.06)	0.848	0.99 (0.97–1.02)	0.456
**Gender n, %**	**Female**	1.193 (0.526–2.703)	0.673	0.893 (0.563–1.415)	0.629	0.57 (0.27–1.17)	0.126	1.26 (0.85–1.87)	0.254
**BMI, kg/m** ^**2**^		0.942 (0.861–1.031)	0.192	1.020 (0.976–1.066)	0.374	1.00 (0.93–1.07)	0.943	1.04 (1.00–1.08)	0.062
**Medical History**									
**Smoker**	**Yes**	1.090 (0.371–3.205)	0.875	0.715 (0.356–1.436)	0.345	0.73 (0.26–2.09)	0.561	0.90 (0.51–1.58)	0.707
**Prior Atrial fibrillation**	**Yes**	0.486 (0.165–1.429)	0.190	1.246 (0.768–2.021)	0.372	1.23 (0.59–2.55)	0.577	N/A	N/A
**Peripheral Vascular Disease (PVD)**	**Yes**	2.149 (0.847–5.451)	0.107	1.050 (0.553–1.994)	0.882	0.39 (0.09–1.65)	0.201	0.88 (0.49–1.57)	0.660
**Medication**									
**Insulin**	**Yes**	0.982 (0.228–4.234)	0.981	1.567 (0.772–3.180)	0.213	0.99 (0.30–3.25)	0.982	1.16 (0.60–2.24)	0.666
**Oral diabetic**	**Yes**	0.463 (0.155–1.384)	0.168	0.914 (0.536–1.561)	0.743	0.37 (0.14–0.96)	0.041	0.81 (0.51–1.29)	0.371
**Statins**	**Yes**	1.679 (0.571–4.936)	0.346	0.729 (0.447–1.187)	0.204	1.26 (0.55–2.92)	0.587	1.10 (0.69–1.77)	0.686
**Statin intensity (only among users)**									
**High vs Low/Moderate**	**Yes**	1.838 (0.723–4.667)	0.201	**3.044 (1.614–5.740)**	**0.001**	0.83 (0.38–1.84)	0.651	**1.61 (1.02–2.54)**	**0.042**
**Laboratory**									
**White blood cells (K/uL)**		**1.153 (1.051–1.266)**	**0.003**	0.960 (0.904–1.019)	0.178	1.03 (0.94–1.12)	0.569	1.00 (0.95–1.05)	0.989
**Total cholesterol (mg/dl)**		0.988 (0.976–1.000)	0.054	**0.993 (0.987–0.999)**	**0.049**	1.00 (0.99–1.01)	0.739	1.00 (0.99–1.00)	0.460
**Creatinine (mg/dl)**		0.993 (0.593–1.664)	0.979	1.148 (0.935–1.411)	0.188	0.46 (0.14–1.50)	0.199	0.90 (0.66–1.22)	0.477
**Echocardiography**									
**Septum thickness (mm)**		**1.161 (1.023–1.317)**	**0.021**	0.954 (0.904–1.007)	0.090	1.05 (0.96–1.15)	0.296	1.02 (0.97–1.08)	0.396
**LVEF%**		1.001 (0.959–1.046)	0.946	0.988 (0.966–1.011)	0.306	1.03 (0.99–1.08)	0.139	1.01 (0.98–1.03)	0.597
**Aortic valve** Mean **gradient** **(mm Hg)**		**1.026 (1.012–1.040)**	**0.003**	0.995 (0.988–1.003)	0.199	1.00 (0.99–1.02)	0.452	1.01 (1.00–1.01)	0.065
**Aortic valve area(cm²)**		0.541 (0.072–4.034)	0.549	**0.177 (0.052–0.604)**	**0.006**	0.79 (0.16–3.79)	0.764	1.06 (0.45–2.45)	0.899
**Procedural parameters**									
**Valve type**	**BEV vs. SEV**	1.148 (0.487–2.709)	0.752	1.070 (0.657–1.744)	0.787	1.48 (0.73–3.00)	0.276	**1.60 (1.07–2.39)**	**0.021**
**Valve size**		0.965 (0.834–1.117)	0.634	0.949 (0.870–1.036)	0.242	0.95 (0.84–1.08)	0.466	**0.93 (0.86–1.00)**	**0.049**
**Post dilatation**	**Yes**	1.765 (0.774–4.025)	0.177	1.031 (0.629–1.689)	0.904	1.19 (0.57–2.46)	0.643	1.00 (0.65–1.55)	0.993
**Procedural time (m)**		**1.013 (1.004–1.023)**	**0.004**	0.996 (0.987–1.005)	0.333	1.01 (1.00–1.02)	0.076	0.99 (0.98–1.00)	0.054
**Electrocardiography & CT**									
**QTc Interval (ms)**		1.011 (0.997–1.024)	0.126	0.999 (0.991–1.006)	0.721	1.00 (0.99–1.01)	0.861	1.00 (0.99–1.01)	0.762
**RBBB**	**Yes**	N/A	N/A	**3.499 (1.411–8.679)**	**0.007**	1.30 (0.18–9.51)	0.797	0.79 (0.20–3.21)	0.744
**LBBB**	**Yes**	1.934 (0.261–14.351)	0.519	0.566 (0.079–4.074)	0.572	N/A	N/A	0.40 (0.06–2.89)	0.366
**LAHB**	**Yes**	2.280 (0.847–6.141)	0.103	0.708 (0.307–1.631)	0.417	1.16 (0.41–3.30)	0.785	1.36 (0.77–2.40)	0.285
**Annulus area (mm2)**		**1.004 (1.001–1.008)**	**0.006**	1.000 (0.998–1.001)	0.681	1.00 (1.00–1.00)	0.325	1.00 (1.00–1.00)	0.634
**Annulus perimeter (mm)**		**1.045 (1.013–1.078)**	**0.005**	0.999 (0.987–1.010)	0.808	1.00 (0.99–1.02)	0.709	1.00 (0.99–1.01)	0.979

**Fig 2:**
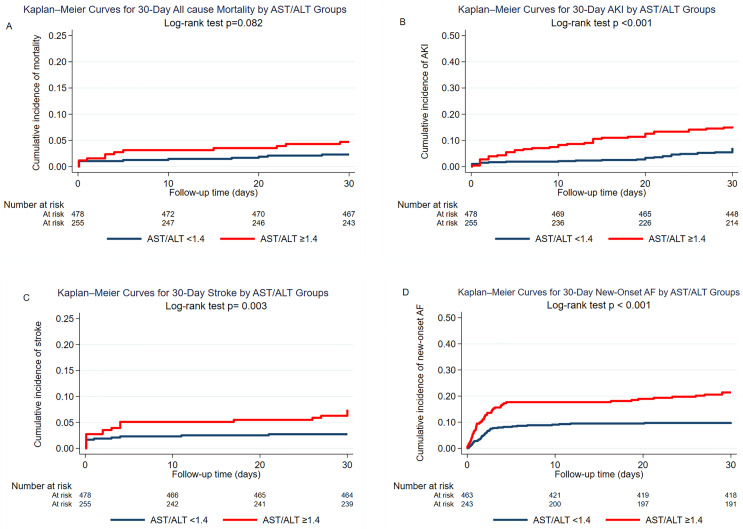
Kaplan–Meier Curves for 30-Day Outcomes According to Preprocedural AST/ALT Ratio (≥1.4 vs < 1.4). (A) All-cause mortality; (B) Acute kidney injury; (C) Stroke; (D) New-onset atrial fibrillation (NOAF).

Kaplan–Meier analyses demonstrated clear early divergence between groups for AKI, stroke, and NOAF, with log-rank *p* < 0.01 for each. No significant separation was observed for mortality (log-rank *p* = 0.18). Cumulative 30-day event rates were approximately threefold higher in the high-ratio group for all secondary outcomes (Fig 2).

In crude Cox analyses, a higher AST/ALT ratio (≥1.4) was associated with increased 30-day risk of acute kidney injury (HR 2.29; 95% CI 1.45–3.63; p < 0.001), stroke (HR 2.77; 95% CI 1.37–5.61; p = 0.004), and NOAF (HR 2.30; 95% CI 1.54–3.41; p < 0.001), whereas the crude association with all-cause mortality did not reach statistical significance (HR 2.07; 95% CI 0.91–4.68; p = 0.082) (Table 3).

**Table 3:** Crude and Adjusted Associations Between Preprocedural AST/ALT Ratio and 30-Day Outcomes After TAVR.

AST/ALT Ratio groups	TAVR-All cause mortality ^a^ (n = 733, events = 23)		TAVR-AKI^b^ (n = 733, events = 73) for crude (n = 537, events = 49) for adjusted		TAVR-Stroke^c^ (n = 733, events = 32)		TAVR-NOAF^d^ (n = 513, events = 97)	
	HR (%95 CI)	P value	HR (%95 CI)	P value	HR (%95 CI)	P value	HR (%95 CI)	P value
***Crude***								
**Low AST/ALT Ratio**	**Ref**							
**High AST/ALT Ratio**	2.07 (0.91–4.68)	0.082	**2.29 (1.45–3.63)**	**<0.001**	**2.77 (1.37–5.61)**	**0.004**	**2.30(1.54–3.41)**	**< 0.001**
***Adjusted***								
**Low AST/ALT Ratio**	Ref							
**High AST/ALT Ratio**	1.74 (0.75–4.04)	0.197	**3.18 (1.80–5.63)**	**<0.001**	**2.73 (1.31–5.69)**	**0.008**	**2.35 (1.57–3.51)**	**<0.001**

**Values are derived from crude and adjusted Cox proportional hazards regression analyses. HR indicates hazard ratio; CI, confidence interval; BEV, balloon-expandable valve; SEV, self-expanding valve; LBBB, left bundle branch block; RBBB, right bundle branch block; LAHB, left anterior hemiblock; PPI, permanent pacemaker implantation; TAVR, transcatheter aortic valve replacement. ᵃ adjusted for age, WBC, aortic valve mean gradient, interventricular septum thickness, procedural time, aortic annulus area, and aortic annulus perimeter.** ^**b**^ **adjusted for statin intensity, total cholesterol, aortic valve area, and RBBB.** ^**c**^**Adjusted for oral antidiabetic therapy, procedural time, baseline creatinine, and mean aortic valve gradient.** ^**d**^ **Adjusted for AST/ALT ratio group (≥1.4 vs < 1.4), age, sex, BMI, baseline creatinine, post-dilatation, procedural time, valve type, CRBBB, aortic annulus perimeter, and QTc interval.**

After adjustment for covariates identified by LASSO and clinical relevance, a higher AST/ALT ratio (≥1.4) remained independently associated with increased 30-day risk of acute kidney injury (adjusted HR 3.18; 95% CI 1.80–5.63; *p* < 0.001), stroke (adjusted HR 2.73; 95% CI 1.31–5.69; *p* = 0.008), and new-onset atrial fibrillation (adjusted HR 2.35; 95% CI 1.57–3.51; *p* < 0.001) whereas the association with all-cause mortality remained non-significant after adjustment (adjusted HR 1.74; 95% CI 0.75–4.04; p = 0.197) (Table 3). Full multivariable models are presented in S1 File

In sensitivity analyses modeling the AST/ALT ratio as a continuous variable per 1-standard deviation increase (SD = 0.67), higher AST/ALT values were significantly associated with all examined 30-day outcomes, including all-cause mortality (adjusted HR 1.31, 95% CI 1.07–1.60, p = 0.010), acute kidney injury (adjusted HR 1.22, 95% CI 1.03–1.45, p = 0.021), stroke (adjusted HR 1.31, 95% CI 1.06–1.62, p = 0.011), and new-onset atrial fibrillation (adjusted HR 1.27, 95% CI 1.08–1.49, p = 0.004). These findings were consistent with the primary categorical analysis and further support a graded association between AST/ALT ratio and adverse outcomes. Detailed results are provided in S1 File (eTable 5).Detailed results of the sensitivity analysis including patients with extreme AST/ALT values are presented in S1 File (eTable 6).

To further explore whether the association between AST/ALT ratio and early adverse outcomes was modified by right-sided disease, we performed exploratory analyses using available echocardiographic surrogates, including moderate/severe tricuspid regurgitation (TR ≥ 2) and pulmonary hypertension (PHT ≥ 40 mmHg). High AST/ALT ratio was not significantly associated with either TR ≥ 2 or PHT ≥ 40 mmHg at baseline. In exploratory Cox interaction analyses, there was no statistically significant interaction between AST/ALT ratio and TR ≥ 2 or PHT ≥ 40 mmHg for 30-day mortality, stroke, or new-onset atrial fibrillation. The interaction between AST/ALT ratio and TR ≥ 2 for acute kidney injury showed a borderline but non-significant signal. Detailed results are presented in S1 File (eTable 7).

As an additional sensitivity analysis addressing potential temporal confounding, we repeated the endpoint-specific multivariable Cox models with additional adjustment for procedural era, categorized as 2016–2019, 2020–2022, and 2023–2025. After adjustment for procedural era, high AST/ALT ratio remained significantly associated with 30-day acute kidney injury, stroke, and new-onset atrial fibrillation. The era-adjusted mortality model was not reported because the small number of deaths and sparse event distribution across procedural eras resulted in unstable estimates. Detailed results are presented in S1 File (eTable 8).

Available antithrombotic medication variables were also summarized according to AST / ALT ratio group. Aspirin, clopidogrel, any P2Y12 inhibitor, dual antiplatelet therapy, oral anticoagulant use, direct oral anticoagulant use, and combined oral anticoagulant plus antiplatelet therapy were similar between the low and high AST / ALT ratio groups. Detailed results are presented in S1 File (eTable 9).

## Discussion

In this retrospective cohort of patients undergoing TAVR, a higher preprocedural AST/ALT ratio was associated with increased 30-day risks of stroke, NOAF, and acute kidney injury. To our knowledge, this is among the first studies in the TAVR setting to evaluate the preprocedural AST/ALT ratio across multiple clinically relevant early post-procedural outcomes within a unified analytic framework. These findings suggest that the AST/ALT ratio may serve as a candidate risk marker, although the observational design does not permit causal inference or immediate clinical implementation. Importantly, the ≥ 1.4 threshold was adopted from prior cardiovascular literature rather than derived within a TAVR-specific cohort; it should therefore be regarded as a literature-based exploratory cut-off that requires external validation in dedicated TAVR populations.

An important strength of the present analysis is the consistency of findings across both categorical and continuous modeling approaches. While the primary analysis used a prespecified threshold (≥1.4) to enhance clinical interpretability, the additional continuous analysis demonstrated that higher AST/ALT values were associated with adverse outcomes in a graded manner across all endpoints.

The continuous sensitivity analysis also addresses potential threshold dependency related to the prespecified AST/ALT cut-off. Because the association between higher AST/ALT values and adverse outcomes persisted when the biomarker was modeled per 1-standard deviation increase, the findings appear less likely to be explained solely by the dichotomization at 1.4. We did not perform data-driven optimization of alternative cut-offs within this cohort, as such an approach could increase the risk of overfitting and produce unstable thresholds in a single-center retrospective dataset with a limited number of events. Therefore, the 1.4 threshold should be interpreted as a pragmatic, literature-informed cut-off, while the continuous analysis supports a graded risk association that requires external validation.

This observation is consistent with prior cardiovascular literature, which has predominantly evaluated the AST/ALT (De Ritis) ratio as a continuous biomarker and has shown that increasing values are associated with worse outcomes in conditions such as acute myocardial infarction and coronary artery disease [14,16]. These studies support the concept that the AST/ALT ratio reflects systemic metabolic stress, inflammation, and organ vulnerability rather than a strict threshold-dependent effect. Importantly, the concordance between categorical and continuous results in our study suggests that the observed associations are not driven by arbitrary dichotomization, but rather reflect an underlying biological gradient linking systemic stress and multisystem vulnerability to early post-TAVR complications. This interpretation is further supported by mechanistic insights highlighting the role of the De Ritis ratio as an integrative marker of hepatic, cardiovascular, and metabolic stress responses [16]. From a clinical perspective, these findings support the use of the AST/ALT ratio as a pragmatic risk marker that can be interpreted both continuously and using simplified thresholds, depending on the clinical context.

Another relevant consideration is the potential influence of underlying coronary artery disease. Although CAD was reported among baseline characteristics and did not differ significantly between AST/ALT groups, the present analysis did not evaluate CAD severity, anatomical extent, multivessel involvement, prior coronary revascularization, completeness of revascularization, ischemia burden, or subsequent ischemic events. This distinction is important because ischemic events after TAVR are increasingly recognized as clinically relevant and may be influenced by baseline CAD burden, previous revascularization status, and multivessel disease [26]. Therefore, part of the observed association between a higher AST/ALT ratio and early adverse outcomes may reflect broader systemic atherosclerotic burden, ischemic vulnerability, or cardio-metabolic stress rather than a TAVR-specific biological signal alone. Accordingly, the AST/ALT ratio should be interpreted as an integrative marker of systemic vulnerability, and future studies should examine whether its prognostic value persists after more granular adjustment for CAD burden and post-TAVR ischemic events.

Another clinically relevant consideration is the potential influence of post-TAVR antithrombotic therapy. Antithrombotic management after TAVR is central to secondary vascular prevention and may influence early thrombotic and bleeding outcomes. Contemporary evidence indicates that current strategies generally favor single antithrombotic therapy, including single antiplatelet therapy in patients without atrial fibrillation and oral anticoagulation alone in patients with atrial fibrillation, while emphasizing individualized treatment according to patient characteristics, concomitant coronary artery disease, recent coronary intervention, bleeding risk, and other clinical indications [27]. In the present study, available antithrombotic medication variables were summarized descriptively according to AST/ALT ratio group and are presented in S1 File (eTable 9). However, the available data did not fully capture the complexity of post-TAVR antithrombotic management, including treatment indication, timing relative to TAVR, dose, duration, adherence, transitions between regimens, temporary interruption, or bleeding-related discontinuation. Therefore, although the descriptive analysis improves transparency regarding available antithrombotic therapy variables, residual confounding related to post-procedural antithrombotic strategy cannot be excluded. This is particularly relevant because early outcomes such as stroke, myocardial ischemic events, valve thrombosis, bleeding events, and new-onset atrial fibrillation may be influenced by individualized antithrombotic decisions after TAVR.

An additional methodological consideration relates to the applied exclusion criteria and their potential impact on selection bias. In the present study, exclusions were restricted to baseline-defined criteria, including missing baseline AST/ALT data, liver cirrhosis, and extreme AST/ALT values, while outcome-specific analyses were conducted using available data for each endpoint. This approach was adopted to avoid unnecessary cohort-wide exclusions and to better reflect real-world clinical data structure.

The exclusion of deaths occurring within 24 hours after TAVR was based on clinical considerations, as these events were predominantly related to acute procedural or mechanical complications (e.g., annular rupture, cardiac tamponade, or major vascular injury), which are unlikely to be causally related to baseline systemic biomarkers. Therefore, their exclusion was intended to reduce non-biological noise rather than introduce bias.

Furthermore, exclusion of extreme AST/ALT values above the 99th percentile (>3.67) was prespecified prior to outcome analysis to minimize the influence of atypical or non-cardiac causes of enzyme elevation. Importantly, sensitivity analyses including these patients demonstrated consistent results across all outcomes, supporting the robustness of the findings and indicating that the observed associations were not driven by outlier values.

Taken together, these methodological considerations support the internal validity of the study and suggest that the observed associations between the AST/ALT ratio and early post-TAVR outcomes are unlikely to be explained by selection bias related to the applied exclusion criteria.

An elevated AST/ALT ratio reflects greater mitochondrial AST release, systemic inflammatory tone, endothelial dysfunction, and oxidative stress domains that plausibly raise embolic propensity and lower the threshold for blood–brain-barrier failure and hemorrhagic transformation in the immediate post-TAVR period. Consistent with this biology, Xu et al. reported that a higher admission AST/ALT ratio independently predicted worse functional recovery at 1 year after acute stroke [28]. Gao et al. concluded that liver-fibrosis markers incorporating AST/ALT independently predicted hemorrhagic transformation and symptomatic intracranial hemorrhage after endovascular therapy [29]. Against this direction, Duan et al. reviewed hepatic responses to stroke and summarized studies in which higher aminotransferase activity tracked smaller infarcts and better outcomes, suggesting context- and timing-dependent effects along the liver–brain axis [30]. Taken together, these findings support the possibility that a higher preprocedural AST/ALT ratio marks increased early neurologic vulnerability after TAVR. However, the present observational data do not establish mechanism and should not be used in isolation to guide antithrombotic therapy, embolic protection, or neuro-monitoring strategies.

From a systems-physiology lens, a higher AST/ALT may index cardio-hepatic congestion, global hypoperfusion, and multisystem mitochondrial stress. Nevertheless, in our cohort, the association with mortality was not significant. Congruently, Ndrepepa et al., who observed that when aminotransferases were within a healthy range, the De Ritis ratio was not independently associated with 3-year all-cause mortality (adjusted HR 1.16, 95% CI 0.94–1.42, p = 0.159) [17]. Similarly, Yang et al. reported a non-linear relationship in critically ill older adults in which the risk was not statistically significant once AST/ALT exceeded the inflection point of 1.80 (HR 1.01, 95% CI 0.96–1.07, p = 0.609), reinforcing that the prognostic signal is context- and range-dependent [31]. These findings suggest that the association between the AST/ALT ratio and mortality may be context-dependent and non-linear. In this setting, the AST/ALT ratio should be interpreted cautiously and as one component of a broader clinical assessment rather than as an independent determinant of mortality risk.

The potential contribution of right-sided dysfunction and venous congestion is also clinically relevant. Because aminotransferase abnormalities may partly reflect cardio-hepatic congestion, we explored whether available right-sided echocardiographic surrogates modified the association between AST/ALT ratio and early adverse outcomes. In these exploratory analyses, high AST/ALT ratio was not significantly associated with moderate/severe tricuspid regurgitation or pulmonary hypertension at baseline, and no statistically significant interaction was observed between AST/ALT ratio and these right-sided surrogates for mortality, stroke, or new-onset atrial fibrillation. A borderline interaction signal was observed for acute kidney injury with tricuspid regurgitation, but this finding was not statistically significant and should be interpreted cautiously given the exploratory nature of the analysis and incomplete availability of detailed right-sided functional data. Overall, these findings suggest that the observed associations were not clearly explained by measured TR or pulmonary hypertension alone, although unmeasured RV dysfunction or venous congestion may still contribute to the AST/ALT risk signal.

Hemodynamic–mitochondrial coupling offers a renal rationale: higher AST/ALT may flag peri-procedural hypoperfusion, oxidative stress, and inflammatory activation that heighten tubular susceptibility to stroke and contrast-mediated injury after TAVR. In coronary cohorts, Zhang et al. found in elective PCI that the De Ritis ratio independently predicted contrast-associated AKI (OR 2.24, 95% CI 1.82–2.76), with an AUC 0.813 and an optimal cut-off 2.97 (sensitivity 67.0%, specificity 82.4%) [32]. Shaik et al. reported that a higher AST/ALT ratio was associated with contrast-associated acute kidney injury in women undergoing elective PCI [33]. Nishino et al. reported that among patients requiring dialysis that higher De Ritis ratios portended worse renal-related outcomes, underscoring its linkage to kidney failure severity [34]. In contrast, Liu et al. found that while AST/ALT tracked short-term death, it did not significantly predict stage 2–3 (“major”) AKI (per-SD HR 1.12, 95% CI 0.99–1.27) in heart-failure inpatients with diabetes [35]. These findings raise the possibility that the AST/ALT ratio reflects broader physiologic vulnerability relevant to renal complications after TAVR. However, prospective validation is required before incorporating this marker into procedural planning or renal-protection pathways.

Electrophysiologically, systemic inflammation, oxidative stress, and neurohumoral activation linked to a higher pre-procedural AST/ALT ratio may promote atrial ectopy, shorten atrial effective refractory periods, and unmask a vulnerable substrate; in our context, periprocedural atrial stretch and autonomic swings around TAVR could further lower the threshold for AF initiation. Consistent with our aim of evaluating early post-TAVR events by AST/ALT strata, the clinical salience of post-TAVR NOAF is supported by Ryan et al., whose meta-analysis of 179 studies (n = 241,712) estimated a pooled NOAF occurrence of 9.9% (95% CI 8.1–12.0), underscoring NOAF as a clinically relevant endpoint for investigation after TAVR [7]. Likewise, Vora et al. (STS/ACC TVT Registry; n = 13,556) observed NOAF in 8.4% overall, with a marked access-route gradient 4.4% after transfemoral versus 16.5% after non-transfemoral approaches illustrating procedural contexts that may interact with biological risk (including higher AST/ALT) and informing our pre-specified adjustments and sensitivity analyses; notably, only 28.9% with NOAF left hospital on oral anticoagulation [36]. By contrast, Nuche et al. implemented systematic ambulatory ECG monitoring and detected largely subclinical NOAF in 7% of recipients, with initiation of anticoagulation in about half, a signal that highlights detection heterogeneity and supports cautious interpretation of NOAF ascertainment across studies [37].

This study has several strengths. First, it evaluates a routinely available laboratory marker in relation to multiple clinically relevant 30-day outcomes after TAVR, with each endpoint analyzed separately rather than as part of a composite outcome. Second, the study used a clearly defined retrospective cohort and multivariable modeling to examine whether the AST/ALT ratio retained an independent association with early post-procedural complications. Third, the assessed outcomes were clinically meaningful and aligned with early post-TAVR follow-up. Finally, because AST and ALT are widely available laboratory measures, the AST/ALT ratio may represent a practical candidate risk marker for future validation studies in broader TAVR populations.

### Limitations

This retrospective, single-center analysis is susceptible to selection and information bias, which may limit its generalizability across programs, device families, and implantation techniques. In addition, because of the observational design, the AST/ALT ratio should be interpreted as a risk marker associated with early adverse outcomes rather than as a mechanistic driver or a basis for direct clinical decision-making. Although we adjusted for major confounders, residual confounding cannot be fully excluded, and additional unmeasured factors, including frailty, inflammatory status, periprocedural hemodynamic instability, and aspects of contrast management, were not fully captured. In addition, although coronary artery disease was included among baseline characteristics, it was captured as a binary comorbidity and was not characterized according to severity, anatomical extent, multivessel involvement, ischemia burden, prior PCI or CABG, completeness of revascularization, or post-TAVR ischemic events. Therefore, residual confounding related to underlying CAD burden and ischemic vulnerability cannot be excluded. This is particularly relevant because an elevated AST/ALT ratio may partly reflect systemic atherosclerotic disease, cardio-metabolic stress, or ischemic susceptibility rather than a TAVR-specific pathophysiologic signal.

Procedural heterogeneity represents an additional potential source of residual confounding. Although several procedural variables were included in the descriptive and multivariable analyses, the available data could not fully capture differences in valve platform selection, device generation, implantation technique, imaging guidance, operator experience, peri-procedural management, or post-procedural care. These factors may independently influence early outcomes such as stroke, acute kidney injury, and new-onset atrial fibrillation. This issue is particularly relevant because the study period extended from 2016 to 2025, during which TAVR practice evolved substantially with respect to device technology, preprocedural imaging, procedural standardization, vascular access management, renal-protection strategies, rhythm monitoring, and early follow-up. To partially address temporal confounding, we performed an additional sensitivity analysis adjusting for procedural era, categorized as 2016–2019, 2020–2022, and 2023–2025. In these era-adjusted models, the associations between high AST/ALT ratio and 30-day acute kidney injury, stroke, and new-onset atrial fibrillation remained consistent with the primary analyses, whereas the mortality model was not reported because the limited number of deaths and sparse event distribution across procedural eras resulted in unstable estimates. These results are presented in S1 File (eTable 8). Nevertheless, procedural-era adjustment cannot fully account for unmeasured changes in TAVR practice over time. Therefore, residual confounding related to procedural heterogeneity and temporal evolution in TAVR practice cannot be excluded and should be considered when interpreting the observed associations.

Although exploratory analyses were performed using available right-sided echocardiographic surrogates, including tricuspid regurgitation and pulmonary hypertension, detailed assessment of right-sided cardiac function and venous congestion was limited. Quantitative right ventricular functional parameters, right atrial pressure estimates, inferior vena cava indices, and directly measured central venous pressure were not systematically available with sufficient completeness. Therefore, we could not fully determine whether the association between AST/ALT ratio and early adverse outcomes was independent of right-sided dysfunction or whether it partly reflected an unmeasured cardio-hepatic congestion phenotype. Future studies should evaluate the AST/ALT ratio alongside detailed RV function, pulmonary pressures, right atrial pressure, inferior vena cava parameters, and invasive hemodynamic measurements, and should formally test whether right-sided dysfunction modifies the prognostic value of AST/ALT after TAVR.

Another limitation is the incomplete characterization of post-TAVR antithrombotic therapy. Although available antithrombotic medication use was descriptively assessed, the dataset did not systematically capture treatment indication, timing relative to TAVR, dose, duration, adherence, treatment transitions, temporary interruptions, combination strategy, or bleeding-related discontinuation. This is relevant because early outcomes such as stroke, ischemic events, valve thrombosis, bleeding, and new-onset atrial fibrillation may be influenced by post-TAVR antithrombotic management, which may have evolved over the study period and been individualized according to atrial fibrillation, coronary artery disease, prior revascularization, bleeding risk, and other clinical indications. Therefore, residual confounding related to post-procedural antithrombotic therapy cannot be excluded.

Competing-risk considerations are also relevant, particularly for nonfatal outcomes such as AKI, stroke, and NOAF, because early death may preclude subsequent event ascertainment. In addition, NOAF detection may have been influenced by the intensity and duration of in-hospital rhythm monitoring, electrocardiographic documentation, and variability in post-discharge surveillance, raising the possibility of detection bias. In addition, although patients with liver cirrhosis and extreme aminotransferase elevations were excluded, milder or subclinical hepatic conditions were not systematically accounted for. Therefore, residual hepatic confounding cannot be fully excluded, as variations in AST/ALT levels may partially reflect underlying liver pathology rather than solely systemic or cardiovascular risk.

Another important limitation is the exclusive focus on 30-day outcomes. Although early complications after TAVR are clinically relevant, a one-month follow-up period is insufficient to determine whether the AST/ALT ratio reflects stable long-term cardiovascular vulnerability or primarily captures transient peri-procedural risk. Outcomes such as acute kidney injury, stroke, and new-onset atrial fibrillation may be strongly influenced by procedural factors, peri-hospitalization management, hemodynamic instability, contrast exposure, antithrombotic strategy, rhythm-monitoring intensity, and early post-discharge surveillance. Therefore, the observed associations should be interpreted as reflecting short-term vulnerability during the early post-TAVR period rather than established long-term prognostic significance. Longer follow-up studies are needed to determine whether the AST/ALT ratio predicts late cardiovascular events, recurrent complications, or long-term mortality after TAVR.

In addition, although the AST/ALT ratio was analyzed using both categorical and continuous approaches, the selected threshold of 1.4 was informed by prior literature and was not specifically derived or externally validated within a TAVR population. We did not perform data-driven optimization of alternative cut-offs in this cohort because such analyses may be unstable in a single-center retrospective dataset and require external validation. Therefore, this cut-off should be interpreted as a pragmatic exploratory threshold, and future studies should evaluate the shape of the risk relationship across the full AST/ALT distribution using larger cohorts and risk-adjusted graphical or spline-based approaches.

## Conclusion

In this retrospective cohort of patients undergoing TAVR, a higher preprocedural AST/ALT ratio was independently associated with increased 30-day risks of acute kidney injury, stroke, and new-onset atrial fibrillation, but not with 30-day all-cause mortality after multivariable adjustment. These findings support the AST/ALT ratio as a candidate marker of early post-TAVR vulnerability. However, the short follow-up period and observational design preclude causal inference and do not allow assessment of long-term prognostic significance. External validation with longer follow-up is required before clinical implementation.

## Supporting information

S1 File Multivariable Cox regression models for 30-day outcomes after TAVR. This file contains the full multivariable Cox regression models for 30-day all-cause mortality, acute kidney injury, stroke, and new-onset atrial fibrillation.
(DOCX)
