# Supplementary material for: De Ritis (AST/ALT) ratio as a predictor of early adverse outcomes following transcatheter aortic valve replacement
Source: PLoS One. 2026 Jul 28;21(7):e0354267. doi: 10.1371/journal.pone.0354267 (PMC13411933; doi:10.1371/journal.pone.0354267)
Supplement: S1 File — This file contains the full multivariable Cox regression models for 30-day all-cause mortality, acute kidney injury, stroke, and new-onset atrial fibrillation. (DOCX) [file pone.0354267.s001.docx]

**S1 File**

**De Ritis (AST/ALT) Ratio as a Predictor of Early Adverse Outcomes Following Transcatheter Aortic Valve Replacement**

**Supplementary Tables**

This supplementary file provides the full multivariable Cox regression models and additional sensitivity, exploratory, procedural-era, and descriptive medication analyses corresponding to the main manuscript.

**Contents**

• eTable 1. Multivariable Cox Regression for 30-Day All-Cause Mortality After TAVR

• eTable 2. Multivariable Cox Regression for 30-Day Acute Kidney Injury (AKI) After TAVR

• eTable 3. Multivariable Cox Regression for 30-Day Stroke After TAVR

• eTable 4. Multivariable Cox Regression for 30-Day New-Onset Atrial Fibrillation (NOAF) After TAVR

• eTable 5. Sensitivity Analysis Modeling Continuous AST/ALT Ratio (per 1-SD Increase) and 30-Day Outcomes After TAVR

• eTable 6. Sensitivity Analysis Re-including the 8 Patients With Extreme AST/ALT Values (Expanded Overall Cohort, N=741): Continuous AST/ALT Ratio per 1-SD Increase and 30-Day Outcomes After TAVR

• eTable 7. Exploratory Association and Interaction Analyses Between AST/ALT Ratio and Right-Sided Echocardiographic Surrogates

• eTable 8. Sensitivity Analysis Additionally Adjusted for Procedural Era

• eTable 9. Available Antithrombotic Therapy Variables According to AST/ALT Ratio Group

**eTable 1. Multivariable Cox Regression for 30-Day All-Cause Mortality After TAVR**

| **Variable** | **Category / Unit** | **HR (95% CI)** | **P value** |
| --- | --- | --- | --- |
| Age | Per 1-year increase | 1.08 (1.01–1.16) | 0.029 |
| White blood cell count | Per 1 K/µL increase | 1.09 (0.96–1.23) | 0.207 |
| Mean aortic valve gradient | Per 1 mmHg increase | 1.03 (1.01–1.05) | 0.002 |
| Septal thickness | Per 1 mm increase | 1.00 (0.85–1.18) | 0.955 |
| Procedural time | Per 1 min increase | 1.01 (1.00–1.02) | 0.037 |
| Aortic annulus area | Per 1 mm² increase | 1.00 (0.99–1.01) | 0.874 |
| Aortic annulus perimeter | Per 1 mm increase | 1.05 (0.97–1.13) | 0.240 |
| AST/ALT ratio group | ≥1.4 vs <1.4 | 1.74 (0.75–4.04) | 0.197 |

**eTable 2. Multivariable Cox Regression for 30-Day Acute Kidney Injury (AKI) After TAVR**

| **Variable** | **Category / Unit** | **HR (95% CI)** | **P value** |
| --- | --- | --- | --- |
| AST/ALT ratio group | ≥1.4 vs <1.4 | 3.18 (1.80–5.63) | <0.001 |
| Statin intensity | High vs low/moderate | 3.51 (1.75–7.06) | <0.001 |
| Total cholesterol | Per 1 mg/dL increase | 1.00 (0.99–1.01) | 0.898 |
| Aortic valve area | Per 1 cm² increase | 0.21 (0.04–1.01) | 0.051 |
| Complete RBBB | Yes vs No | 3.77 (1.49–9.57) | 0.005 |

**eTable 3. Multivariable Cox Regression for 30-Day Stroke After TAVR**

| **Variable** | **Category / Unit** | **HR (95% CI)** | **P value** |
| --- | --- | --- | --- |
| AST/ALT ratio group | ≥1.4 vs <1.4 | 2.73 (1.31–5.69) | 0.008 |
| Oral antidiabetic therapy | Yes vs No | 0.38 (0.14–0.99) | 0.047 |
| Procedural time | Per 1 min increase | 1.01 (0.99–1.02) | 0.112 |
| Serum creatinine | Per 1 mg/dL increase | 0.44 (0.12–1.57) | 0.207 |
| Mean aortic valve gradient | Per 1 mmHg increase | 1.01 (1.00–1.03) | 0.105 |

**eTable 4. Multivariable Cox Regression for 30-Day New-Onset Atrial Fibrillation (NOAF) After TAVR**

| **Variable** | **Category / Unit** | **HR (95% CI)** | **P value** |
| --- | --- | --- | --- |
| AST/ALT ratio group | ≥1.4 vs <1.4 | 2.35 (1.57–3.51) | <0.001 |
| Age | Per 1 year increase | 1.00 (0.97–1.03) | 0.810 |
| Female sex | Female vs male | 1.19 (0.78–1.81) | 0.418 |
| Body mass index | Per 1 kg/m² increase | 1.04 (1.00–1.08) | 0.077 |
| Serum creatinine | Per 1 mg/dL increase | 0.84 (0.62–1.15) | 0.283 |
| Post-dilatation | Yes vs No | 1.09 (0.68–1.73) | 0.732 |
| Procedural time | Per 1 min increase | 0.99 (0.98–1.00) | 0.033 |
| Valve type | SEV vs BEV | 1.54 (0.99–2.39) | 0.054 |
| Complete RBBB | Yes vs No | 0.68 (0.16–2.85) | 0.602 |
| Aortic annulus perimeter | Per 1 mm increase | 1.00 (0.99–1.01) | 0.932 |
| QTc interval | Per 1 ms increase | 1.00 (0.99–1.01) | 0.960 |

**Abbreviations: AKI, acute kidney injury; ALT, alanine aminotransferase; AST, aspartate aminotransferase; BEV, balloon-expandable valve; CI, confidence interval; HR, hazard ratio; NOAF, new-onset atrial fibrillation; QTc, corrected QT interval; RBBB, right bundle branch block; SEV, self-expanding valve; TAVR, transcatheter aortic valve replacement.**

**eTable 5. Sensitivity Analysis Modeling Continuous AST/ALT Ratio (per 1-SD Increase) and 30-Day Outcomes After TAVR**

| **Outcome** | **Crude HR (95% CI)** | **P value** | **Adjusted HR (95% CI)** | **P value** |
| --- | --- | --- | --- | --- |
| All-cause mortality | 1.29 (1.09–1.53) | 0.003 | 1.31 (1.07–1.60) | 0.010 |
| Acute kidney injury (AKI) | 1.17 (1.02–1.35) | 0.026 | 1.22 (1.03–1.45) | 0.021 |
| Stroke | 1.23 (1.03–1.47) | 0.023 | 1.31 (1.06–1.62) | 0.011 |
| New-onset atrial fibrillation (NOAF) | 1.22 (1.05–1.41) | 0.011 | 1.27 (1.08–1.49) | 0.004 |

**Values are derived from Cox proportional hazards regression analyses modeling the AST/ALT ratio as a continuous variable scaled per 1-standard deviation increase (SD = 0.67). HR indicates hazard ratio; CI, confidence interval; TAVR, transcatheter aortic valve replacement. All-cause mortality was adjusted for age, WBC, aortic valve mean gradient, interventricular septum thickness, procedural time, aortic annulus area, and aortic annulus perimeter. Acute kidney injury (AKI) was adjusted for statin intensity, total cholesterol, aortic valve area, and right bundle branch block (RBBB). Stroke was adjusted for oral antidiabetic therapy, procedural time, baseline creatinine, and mean aortic valve gradient. New-onset atrial fibrillation (NOAF) was adjusted for age, sex, body mass index (BMI), baseline creatinine, post-dilatation, procedural time, valve type, complete right bundle branch block (CRBBB), aortic annulus perimeter, and QTc interval.**

**eTable 6. Sensitivity Analysis Re-including the 8 Patients With Extreme AST/ALT Values (Expanded Overall Cohort, N=741): Continuous AST/ALT Ratio per 1-SD Increase and 30-Day Outcomes After TAVR**

| **Outcome** | **Model** | **HR (95% CI)** | **P value** |
| --- | --- | --- | --- |
| 30-day all-cause mortality | Crude | 1.12 (0.96–1.30) | 0.140 |
|  | Adjusted* | 1.29 (1.05–1.57) | 0.014 |
| Acute kidney injury (AKI) | Crude | 1.04 (0.92–1.17) | 0.508 |
|  | Adjusted† | 1.22 (1.03–1.45) | 0.021 |
| Stroke | Crude | 1.08 (0.92–1.26) | 0.336 |
|  | Adjusted‡ | 1.17 (0.97–1.41) | 0.096 |
| New-onset atrial fibrillation (NOAF) | Adjusted§ | 1.27 (1.08–1.49) | 0.004 |

**Values are derived from Cox proportional hazards regression analyses modeling the AST/ALT ratio as a continuous variable per 1-standard deviation increase (SD = 0.67).**

***Adjusted for age, WBC, aortic valve mean gradient, interventricular septum thickness, procedural time, aortic annulus area, and aortic annulus perimeter.**

**†Adjusted for statin intensity, total cholesterol, aortic valve area, and right bundle branch block (RBBB).**

**‡Adjusted for oral antidiabetic therapy, procedural time, baseline creatinine, and aortic valve mean gradient.**

**§Adjusted for age, sex, body mass index (BMI), baseline creatinine, post-dilatation, procedural time, valve type, complete right bundle branch block (CRBBB), aortic annulus perimeter, and QTc interval.**

**This sensitivity analysis includes patients with extreme AST/ALT values (>99th percentile), which were excluded from the primary analysis.**

**eTable 7. Exploratory Association and Interaction Analyses Between AST/ALT Ratio and Right-Sided Echocardiographic Surrogates**

| **Analysis** | **Right-sided surrogate** | **Result** | **P value** |
| --- | --- | --- | --- |
| Baseline association | Moderate-to-severe tricuspid regurgitation (TR ≥2) | 48.1% vs 44.4% | 0.374 |
|  | Pulmonary hypertension (PHT ≥40 mmHg) | 83.4% vs 84.4% | 0.791 |
| Interaction analysis: 30-day mortality | AST/ALT × moderate-to-severe TR | HR 2.57 (95% CI 0.31–21.12) | 0.379 |
|  | AST/ALT × PHT ≥40 mmHg | HR 0.60 (95% CI 0.02–17.07) | 0.762 |
| Interaction analysis: 30-day AKI | AST/ALT × moderate-to-severe TR | HR 4.09 (95% CI 0.96–17.36) | 0.056 |
|  | AST/ALT × PHT ≥40 mmHg | HR 2.60 (95% CI 0.35–19.62) | 0.353 |
| Interaction analysis: 30-day stroke | AST/ALT × moderate-to-severe TR | HR 3.07 (95% CI 0.54–17.61) | 0.208 |
|  | AST/ALT × PHT ≥40 mmHg | HR 0.83 (95% CI 0.06–11.62) | 0.892 |
| Interaction analysis: 30-day NOAF | AST/ALT × moderate-to-severe TR | HR 1.66 (95% CI 0.69–4.00) | 0.258 |
|  | AST/ALT × PHT ≥40 mmHg | HR 1.85 (95% CI 0.43–7.94) | 0.408 |

**Abbreviations: AKI, acute kidney injury; AST/ALT, aspartate aminotransferase-to-alanine aminotransferase ratio; CI, confidence interval; HR, hazard ratio; NOAF, new-onset atrial fibrillation; PHT, pulmonary hypertension; TR, tricuspid regurgitation.**

**Note: Moderate-to-severe TR was defined as TR grade ≥2. Pulmonary hypertension was defined as PHT ≥40 mmHg using available echocardiographic pulmonary pressure data. These variables were used as exploratory surrogates of right-sided disease and pulmonary pressure burden. Interaction analyses were performed using Cox proportional-hazards models with the same endpoint-specific covariate structure used in the primary adjusted models. These analyses were exploratory and limited by incomplete availability of detailed right ventricular functional parameters, right atrial pressure estimates, inferior vena cava indices, and directly measured central venous pressure.**

**eTable 8. Sensitivity Analysis Additionally Adjusted for Procedural Era**

| **Outcome** | **Exposure** | **Adjusted HR** | **95% CI** | **P value** |
| --- | --- | --- | --- | --- |
| 30-day acute kidney injury | High AST/ALT ratio | 3.15 | 1.78–5.59 | <0.001 |
| 30-day stroke | High AST/ALT ratio | 2.74 | 1.31–5.73 | 0.007 |
| 30-day new-onset atrial fibrillation | High AST/ALT ratio | 2.35 | 1.57–3.52 | <0.001 |

**Abbreviations: AST/ALT, aspartate aminotransferase-to-alanine aminotransferase ratio; CI, confidence interval; HR, hazard ratio; NOAF, new-onset atrial fibrillation.**

**Note: Procedural era was categorized as 2016–2019, 2020–2022, and 2023–2025. Models were adjusted using the same endpoint-specific covariate structure as the primary adjusted analyses, with additional adjustment for procedural era. The 30-day mortality model was not reported in this sensitivity table because the small number of deaths and sparse distribution across procedural eras resulted in unstable era-adjusted estimates.**

**eTable 9. Available Antithrombotic Therapy Variables According to AST/ALT Ratio Group**

| **Antithrombotic variable** | **Low AST/ALT ratio (n=478)** | **High AST/ALT ratio (n=255)** | **P value** |
| --- | --- | --- | --- |
| Aspirin | 310 (64.9%) | 162 (63.5%) | 0.721 |
| Clopidogrel | 278 (58.2%) | 145 (56.9%) | 0.735 |
| Any P2Y12 inhibitor | 283 (59.2%) | 146 (57.3%) | 0.610 |
| Dual antiplatelet therapy | 278 (58.2%) | 145 (56.9%) | 0.735 |
| Any oral anticoagulant | 317 (66.3%) | 164 (64.3%) | 0.586 |
| Any direct oral anticoagulant | 316 (66.1%) | 164 (64.3%) | 0.626 |
| Rivaroxaban | 278 (58.2%) | 145 (56.9%) | 0.735 |
| Apixaban | 286 (59.8%) | 146 (57.3%) | 0.499 |
| Dabigatran | 308 (64.4%) | 163 (63.9%) | 0.890 |
| Warfarin/Coumadin | 279 (58.4%) | 145 (56.9%) | 0.694 |
| Oral anticoagulant plus antiplatelet therapy | 284 (59.4%) | 148 (58.0%) | 0.719 |

**Abbreviations: AST/ALT, aspartate aminotransferase-to-alanine aminotransferase ratio; DOAC, direct oral anticoagulant; OAC, oral anticoagulant; P2Y12, P2Y12 receptor inhibitor.**

**Note: Values are presented as n (%). Medication variables were recoded as binary indicators, with 0 or missing values indicating no documented use and values 1–3 indicating documented use. Derived categories included any P2Y12 inhibitor, dual antiplatelet therapy, any oral anticoagulant, any direct oral anticoagulant, and oral anticoagulant plus antiplatelet therapy. These variables were used for descriptive characterization only, because detailed treatment indication, exact timing relative to TAVR, dose, duration, adherence, treatment transitions, temporary interruptions, and bleeding-related discontinuation were not systematically available.**
